# Topical Application of Sadat-Habdan Mesenchymal Stimulating Peptide (SHMSP) Accelerates Wound Healing in Diabetic Rabbits

**DOI:** 10.1155/2012/531961

**Published:** 2012-06-19

**Authors:** Abdulmohsen H. Al-Elq, Mir Sadat-Ali, Mohamed Elsharawy, Ibrahim Al-Habdan, Fatin Othman Al-Aqeel, Magda M. Naim

**Affiliations:** ^1^Department of Internal Medicine, University of Dammam, Dammam, Saudi Arabia; ^2^King Fahd Hospital of the University, P.O. Box 40071, Al-Khobar 31952, Saudi Arabia; ^3^Department of General Surgery, University of Dammam, Dammam, Saudi Arabia; ^4^Department of Orthopaedic Surgery, University of Dammam, Dammam, Saudi Arabia; ^5^Department of Histology, Suez Canal University, Ismalia, Egypt

## Abstract

*Objective*. Diminished wound healing is a common problem in diabetic patients due to diminished angiogenesis. SHMSP was found to promote angiogenesis. The present study was carried out to examine the effect of this peptide in healing of wounds in diabetic rabbits. *Materials and Methods*. Twenty male New Zealand rabbits were used in this study. Diabetes mellitus was induced and the rabbits were randomly divided into two equal groups: control group and peptide group. A-full thickness punch biopsy was made to create a wound of about 10 mm on the right ears of all rabbits. Every day, the wound was cleaned with saline in control groups. In the peptide group, 15 mg of SHMSP was applied after cleaning. On day 15th, all animals were sacrificed, and the wounds were excised with a rim of 5 mm of normal surrounding tissue. Histo-pathological assessment of wound healing, inflammatory cell infiltration, blood vessel proliferation, and collagen deposition was performed. *Results*. There were no deaths among the groups. There was significant increase in wound healing, blood vessel proliferation and collagen deposition, and significant decrease in inflammatory cell infiltration in the peptide group compared to the control group. *Conclusion*. Topical application of SHMSP improves wound healing in diabetic rabbits.

## 1. Introduction

Impaired wound healing is a common complication of diabetes. Diminished production of growth factors and decreased angiogenesis are thought to contribute to impaired healing of chronic wounds [1]. Angiogenesis is necessary to allow migration of leukocytes and growth factors and oxygen supply during granulation tissue formation. Growth factors and particularly the Vascular Endothelial Growth Factor (VEGF) play an important role in the induction of angiogenesis [2]. Reductions of several growth factors were found to occur in diabetic animals [3]. Application of such growth factors was shown to facilitate wound closure in animals and humans [4–6]. The first approved topically applied growth factor is the recombinant human Platelets Derived Growth Factor (hPDGF-BB). In previous studies, topical application of the hPDGF-BB to acute and chronic wounds resulted in faster complete wound closure than the control wounds [7, 8]. Several other growth factors have been recently used for therapeutic angiogenesis [2].

SHMSP is 13 amino acids peptide, having a molecular weight of 1465 daltons and synthesized in the laboratory. It is absorbed in the body and excreted from the kidney [9]. It was shown to stimulate bone growth and accelerate the healing of bone fracture by way of angiogenesis [10]. On the other hand the rabbit ear ulcer model was found to be favorable animal wound-healing model because it leads to full-thickness noncontractile excisional wound with avascular base [11]. This study was carried out with an objective to evaluate the efficacy of SHMSP in the wound healing of alloxan-induced diabetic rabbits and to study its angiogenesis effect on wounds of such animals.

## 2. Materials and Methods

The experimental study was performed in accordance with the national laws for the principles of laboratory care on animal experiments. It was carried out at the Departments of Medicine, Orthopedic and Surgery, University of Dammam, Saudi Arabia and Histology Department, Faculty of Medicine, Suez Canal University, Egypt in the period between July 2010 and September 2010. Ethical approval had been obtained from the local committee at King Faisal University.

Twenty apparently healthy New Zealand male rabbits, ≥12 weeks old and weighing between 3 and 5 kg, were used in this study. Animals were kept in large cages with free mobility and fed with standard rabbit diet. They were provided with food and water ad libitum and maintained at 25–28°C. Cages were changed routinely every day. Diabetes mellitus was induced by a single intraperitoneal injection of freshly prepared alloxan (Sigma Chemical, USA) at a dose of 160 mg/kg of body weight to overnight fasting animal in total volume of 0.5 mL [12]. After treatment, water containing 1% dextrose was given to rabbits for 48 hours to avoid hypoglycemia. The blood sugar was then measured daily using blood glucose tests strips and meter (ACCU-CHECK, Roche Diagnostics). No rabbit experienced hypoglycemia (<70 mg/dL) or marked hyperglycemia (>600 mg/dL). After one week of having blood sugar of more than 250 mg/dL, rabbits were randomly divided into two equal groups, control group, and peptide treated group (10 rabbits each). A sterile 10-mm diameter punch biopsy was used to create single full-thickness wound on the right ear of each rabbit under anesthesia. Every day, the wound was cleaned with normal saline in the control group while, in the diabetic group, 15 mg of the SHMSP was applied once daily after cleaning to cover the whole wound area. No dressings were applied at the site of the wound throughout the duration of the experiment. On day 15th, all animals were sacrificed, and the wounds' sites were excised with a rim of 5 mm of normal surrounding skin. Tissue specimens were fixed in 10% neutral buffered formalin solution. They were then processed to prepare 5 mm-thick paraffin sections for histological stains (H&E, Masson trichrome and reticulin stains) [13]. Skin sections were assessed by light microscopy for wound healing, inflammatory cell infiltration, blood vessel proliferation, and collagen deposition. Wound healing was assessed in H&E sections. Reticulin stain aimed at detecting early events of wound healing. 

Quantitative analyses of digital images were made with Super eye-Heidi software (Heidi Software Company, Cairo, Egypt). Images were obtained with a CCD video Camera (HS-CC721) mounted on a Microscope (Olympus BH-2). Measurement of the following was performed: (1) area percentage of inflammatory cells in H&E-stained sections, (2) color area percentage of collagen fibers in Masson's trichrome stained sections, (3) vascular area density: percentage of blood vessel area per wound area, and (4) vessel Index (vessels/mm^2^) [14]. Both vascular area density and Vessel Index were measured in Masson's trichrome stained. The image analyzer was calibrated for color and distance measurements before use.

**Statistical Analysis**

Chi square test or Fisher's exact test, was used as appropriate to assess differences in wound healing between groups. Other results were summarized using descriptive statistics. These were presented as mean ± SD and compared using Student *t*-test. Significance was set at *P* < 0.05 for all comparisons. All statistical analyses were performed with the aid of SPSS 15 (SPSS Inc, Chicago, IL, USA) software.

## 3. Results

**Figure 1:**
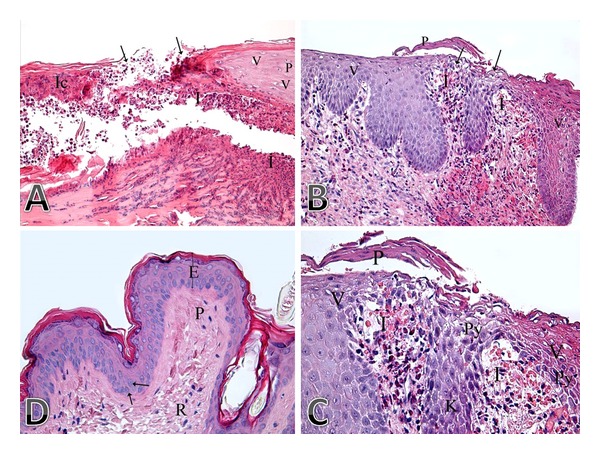
(A, B, and C) Photomicrograph of a skin section from control group showing (A) nonhealed ulcer (arrows). There is dermoepidermal separation due to presence of marked inflammatory cell infiltration (I). The epidermal cells on the left side of the photo are replaced by inflammatory cells (Ic), and those on the right side of the photo show cytoplasmic vacuolation (V) and pyknosis (P) of some of their nuclei. (H&E ×200). (B) Incomplete healing of the ulcer (arrow) due to presence of marked inflammatory cell infiltration (I). The epidermal cells on both sides of the ulcer area show marked increase in thickness (acanthosis), and lots of them appeared vacuolated (V). Note also the presence of parakeratosis (P; presence of nuclei in keratin layer), (H&E ×200). (C) High-power view of the previous photo. There is marked increase in epidermal thickness on both sides of the ulcer area, marked cellular infiltration (I), parakeratosis (P), and vacuolated epidermal cells (V). Some of the epidermal cells also show pyknosis (Py) or karyolysis (K) of their nuclei (H&E ×400). (D) Photomicrograph of a skin section from the peptide group showing complete healing of the ulcer and normal appearance of both the epidermis (E) and dermis (papillary dermis; P and reticular dermis; R). Note also that some of the epidermal cells show mitotic figures (arrow) (H&E ×400).

**Figure 2:**
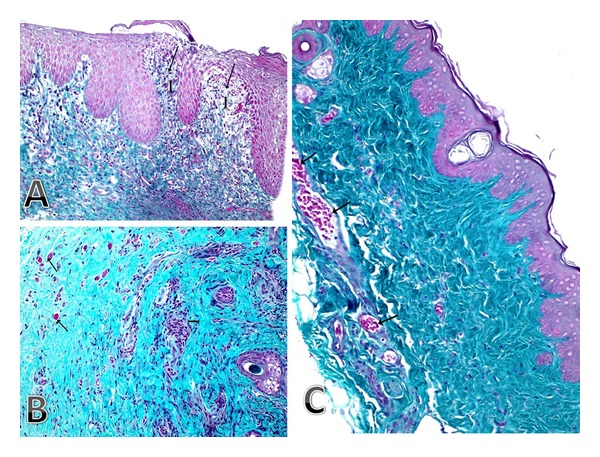
(A and B) Photomicrograph of a skin section from control group showing (A) few collagen fibers under the ulcer (arrow) where the area is replaced by marked inflammatory cell infiltration (I) (Masson's trichrome ×200). (B) An area of the dermis with small and few vasculature (small blood vessels; arrow); however, inflammatory cell infiltration (I) is shown (Masson's trichrome ×200). (C) Photomicrograph of a skin section from the peptide-treated group showing normal appearance of collagen fibers and presence of good vasculature in the dermis (many large blood vessels; arrow) (Masson's trichrome ×200).

**Figure 3:**
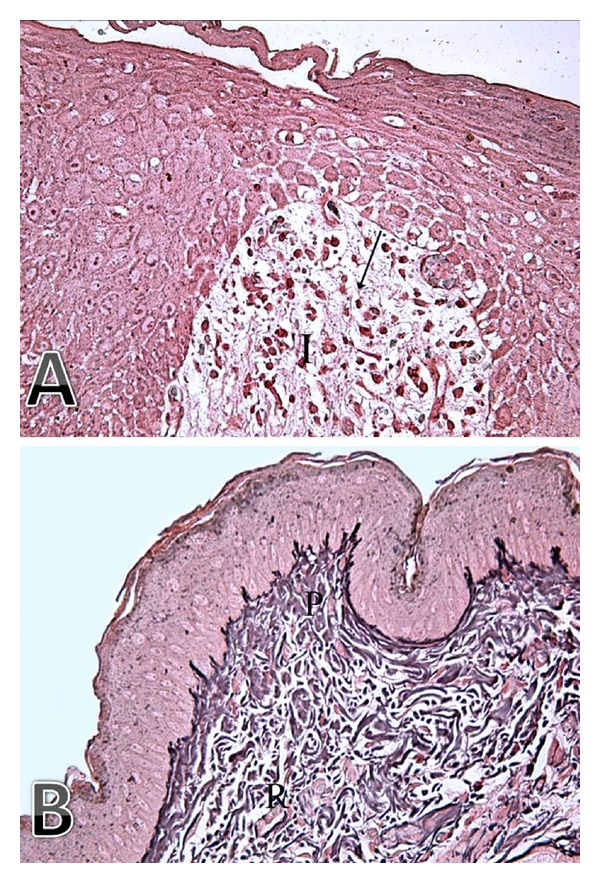
(A) Photomicrograph of a skin section from control group showing loss of reticulin fibers under the ulcer (arrow) where the area is replaced by marked inflammatory cell infiltration (I) (Reticulin stain ×400). (B) Photomicrograph of a skin section from peptide-treated group showing normal appearance of reticular fibers (black colored) in both the papillary (P) and reticular (R) dermis (Reticulin stain ×400).

**Figure 4:**
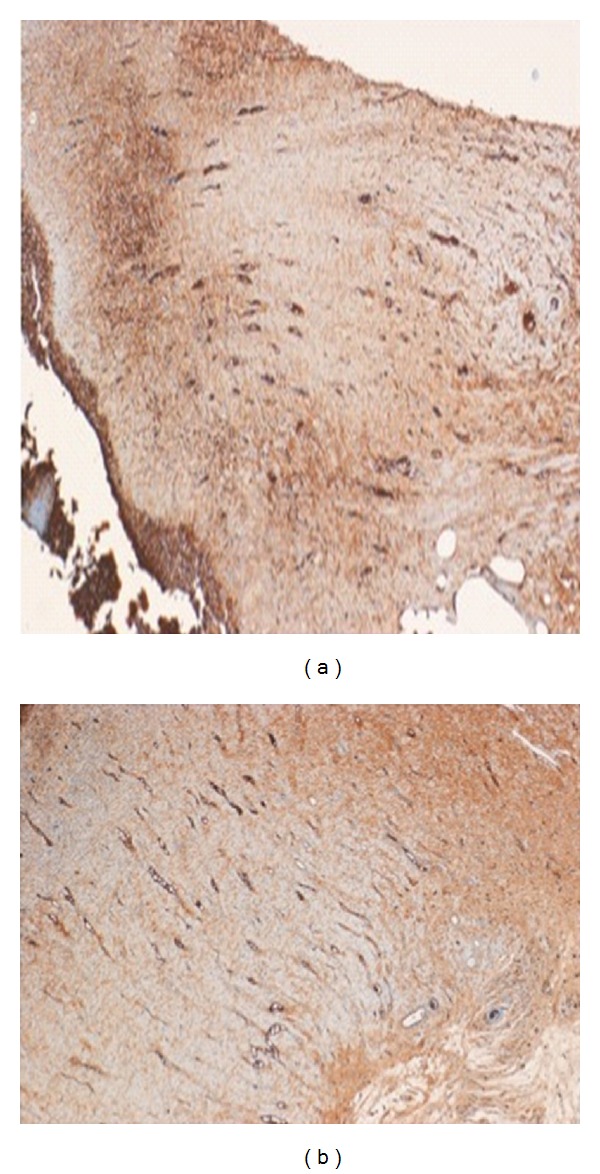
(a) Photomicrograph of a skin section from control group showing few short vascular channels. (b) Photomicrograph of a skin section from peptide-treated group showing widespread long vascular channels (CD31 tissue marker stain, ×100).

All alloxan-treated rabbits developed hyperglycemia within 72 hours. There were no deaths among the groups. Most of the control group showed evidence of nonhealed ulcer and marked inflammatory cell infiltration. The epidermal cells on both sides of the ulcer area showed acanthosis (marked increase in thickness) with cytoplasmic vacuolation, pyknosis, and karyolysis of their nuclei. Parakeratosis (presence of nuclei in keratin layer) was also evident in some cases. Reticulin stain revealed few reticular fibers under the wound. There was also small number of collagen fibers under the ulcer with scattered short blood vessels (Figure 1). On the other hand, the peptide group revealed complete healing of most of the ulcers and normal appearance of both the epidermis and dermis, normal appearance of collagen fibers in both the epidermis and dermis with normal vasculature (Figure 2). Using Reticulin stain, the reticular fibers appeared normal in the peptide-treated group (Figure 3). The growth of new blood vessels was also assessed using CD31 tissue marker stain which revealed widespread and longer vascular channels in the peptide-treated group as compared to the control group (Figure 4).

Quantitative analyses of digital images revealed highly significance lower percentage of inflammatory cells in the treated group as compared to the control (*P* value <0.0001). Collagen deposition as a marker of wound healing was also significantly higher in the treated group. There was clear evidence of enhancement of angiogenesis by SHMSP with significantly higher vessel index (50.13 ± 10.71 versus 26.7 ± 7.44, *P* value <0.0001) and vascular area density in the peptide-treated group as compared to the control group (Table 1).

## 4. Discussion

Diabetes mellitus is increasing in incidence and represents a major health problem for the 21st century. The total number of diabetic patients is expected to increase from 171 million individuals in 2000 to 439 million individuals in 2030 compromising around 7.7% of the world population [15]. One of the main complications of diabetes is foot ulcer affecting from 1.0% to 4.1% of patients while the annual incidence of amputation is 0.21–1.37% [16]. The process of wound healing has three important components: inflammation, proliferation, and tissue remodeling. Inflammatory phase requires functioning immune system. The proliferative phase requires deposition of collagen, and angiogenesis or new vessels formation. During the remodeling phase, reorganization of collagen occurs that leads to restoration of tissue structural integrity [17–19]. In diabetes mellitus these three phases are affected. There is depression of immune system causing increase incidence of wound infection with suppression of angiogenesis and collagen formation [20]. The present study assessed the effect of SHSMP on the above processes during healing of diabetic wounds. 

Within 72 hours, blood sugar measurement indicated the presence of hyperglycemia which persisted during the period of this study. Diabetes mellitus was found in many experimental and clinical studies to increase markers of wound infection manifested by increased inflammatory cells [21–23]. Wound infection is one of the most common causes of delayed wound healing [24], while prevention and treatment of wound infection promotes the healing process [25]. This study revealed that topical application of SHMSP on diabetic wound significantly decreases inflammatory cells and thereby improves healing. It is well established that one of the essential components of normal wound healing is the formation of new blood vessels within the wound matrix referred to as granulation tissue [20, 26]. Diabetes mellitus has been shown to be associated with decrease in number and function of circulating endothelial progenitor cells [27, 28], hence, impaired angiogenesis and wound healing [29, 30]. Our study revealed significant enhancement of angiogenesis in the SHMSP-treated group as compared to the control group. SHMSP was also found earlier to enhance angiogenesis in normoglycemic animals [9]. This study further proved that it could also increase angiogenesis in wounds created in diabetic animals. Any factor which improves angiogenesis in the wound should improve wound healing. Delay in healing of diabetic wound was also reported to be due to impaired fibroblast migration [31]. This will result in reduction of collagen deposition and impediment in wound healing. Reticular fibers are formed mainly of collagen type III which constitutes about 15% of dermal collagen, in association with other types of collagen, glycoprotein (fibronectin), and proteoglycan [32]. It was also reported that fibronectin and collagen type III (reticular fibers) are major constituents of early wound architecture [33]. We think that part of improved wound healing of diabetic rabbits by SHMSP in this study is due to enhancement of collagen deposition and increased reticular fiber formation.

One of mechanisms of VEGF in accelerating experimental diabetic wound healing is stimulating the migration of cultured human keratinocytes and fibroblasts, thus revealing a nonangiogenic effect of VEGF on wound closure [34]. Apparently the same mechanism would apply also for SHMSP which was found in this study to improve collagen deposition, in addition to angiogenesis, and hence promoted diabetic wound healing. Other possible mechanism of improved wound healing by SHMSP in the present study is that this peptide possibly acted like a bone morphogenetic protein (BMP) through stimulation of the basic cellular model at the injury site. It was shown previously that when SHMSP was used in a fracture model, it stimulated osteoblasts formation with deposition of new bone in the early stage of fracture healing [10]. Bone morphogenetic proteins (BMPs) are known to be osteoinductive and stimulated bone formation. Recent reports indicated that BMPs may play a role in angiogenesis as well. Finkenzeller et al. (2012) [35] conclusively showed that BMP-2 stimulates vasculogenesis and angiogenesis. Limitations of this study are the lack of assessment of wound closure and wound volume through imaging and wound tracing and the lack of comparison with finding from baseline skin biopsies. Additional studies should look at the systemic effect of SHMSP and the effect of longer period of hyperglycemia. 

## 5. In Conclusion

Topical application of SHMSP on diabetic wound decreases inflammatory cell infiltration and stimulates blood vessel proliferation and collagen deposition. This can reduce incidence of infection and accelerate wound healing in diabetic rabbits.

## Figures and Tables

**Table 1:** Histological analysis of control and peptide treated groups.

	Control	Peptide treated	*P* value
Healing	2/10	7/10	0.025
Vascular area density (%)	9.37 ± 5.81	21.15 ± 4.04	0.036
Vessel Index (vessels/mm^2^)	26.7 ± 7.44	50.13 ± 10.71	<0.0001
Color area percentage of collagen fibers	35.45 ± 5.82	43.09 ± 6.42	0.013
Area percentage of Inflammatory cell	35.08 ± 15.07	10.02 ± 1.31	<0.0001
